# Construction of Recombinant *Saccharomyces cerevisiae* with Ethanol and Aldehydes Tolerance via Overexpression of Aldehyde Reductase

**DOI:** 10.3390/microorganisms10050850

**Published:** 2022-04-20

**Authors:** Nileema R. Divate, Pei-Ju Huang, Gen-Hung Chen, Yun-Chin Chung

**Affiliations:** 1 Department of Food and Nutrition, Providence University, Taichung 43301, Taiwan; neelima7m@gmail.com (N.R.D.); pelue2002@gmail.com (P.-J.H.); 2 Department of Cosmetic Science, Providence University, Taichung 43301, Taiwan; jhchen2@pu.edu.tw

**Keywords:** *Saccharomyces cerevisiae*, furfural, hydroxy-methyl-furfural, aldehyde reductase, ethanol production

## Abstract

Furfural and hydroxy-methyl-furfural (HMF) are produced by lignocellulosic biomass during heat or acid pretreatment and are toxic to yeast. Aldehyde reductase is the main enzyme to reduce furfural and HMF. To improve the conversion efficiency of lignocellulosic biomass into ethanol, we constructed *Saccharomyces cerevisiae* with overexpression of aldehyde reductase (encoded by *ari1*). The gene of aldehyde reductase (encoded by *ari1*) was cloned via polymerase chain reaction (PCR) and ligated with the expression vector pGAPZαC. Western blot coupled with anti-His tag confirmed overexpression of the *ari1* gene. The growth curves of the wild and *ari1*-overexpressed strain in the YPD medium were found to be almost identical. Compare to the *ari1*-overexpressed strain, the wild strain showed a longer doubling time and lag phase in the presence of 20 mM furfural and 60 mM HMF, respectively. The real-time PCR results showed that furfural was much more potent than HMF in stimulating *ari1* expression, but the cell growth patterns showed that 60 mM HMF was more toxic to yeast than 20 mM furfural. *S. cerevisiae* with *ari1* overexpression appeared to confer higher tolerance to aldehyde inhibitors, thereby increasing the growth rate and ethanol production capacity of *S. cerevisiae* in an aldehyde-containing environment.

## 1. Introduction

Based on the concept of low-cost waste utilization, lignocellulosic biomass and agricultural residues are ideal raw materials for bioethanol production [1,2]. The production of ethanol from lignocellulosic biomass involves the main operations of pretreatment, hydrolysis, fermentation and distillation [3]. Acid hydrolysis or alkali swelling combined with heating is the key technology used to destroy the cellulose crystal structure [4]. Pretreatment of the biomass with acid hydrolysis will produce furfural or hydroxymethyl furfural (HMF), which is the most effective and representative inhibitory compound that interferes with subsequent fermentation [5,6]. After acid/heat treatment, furfural and HMF are formed from the dehydration of pentose and hexose sugars, respectively [7,8]. Research has shown that ethanol productivity in *S. cerevisiae* can be reduced under 30 mM furfural or 60 mM HMF [9]. These inhibitors hinder yeast growth by disrupting cell walls and membranes, reducing enzyme activity, damaging DNA and inhibiting protein and RNA synthesis [9].

*Saccharomyces cerevisiae*, a species of yeast, has been traditionally used for brewing and fuel ethanol production. Additionally, the aldehyde reductase originally present in *S. cerevisiae* can reduce furfural and HMF to furfuryl alcohol [10,11] and 2,5-bis-hydroxymethylfuran [5], respectively. However, in high furfural or HMF environments, *S. cerevisiae* growth is inhibited and, when furfural and HMF are reduced to sufficiently low concentrations, cell growth recovers and ethanol production resumes until fermentation is complete [12,13]. Overexpression of the dehydrogenase/reductase genes *adh6* (encodes for NADPH-dependent Alcohol Dehydrogenase 6), *adh7* (encodes for NADPH-dependent Alcohol Dehydrogenase 7), *ari1* (encodes for NADPH-dependent aldehyde reductase) and *ald6* (encodes for Magnesium-activated aldehyde dehydrogenase) increase furfural and/or HMF reduction and host *S. cerevisiae* strain tolerance to inhibitors [14,15,16,17].

Liu and Moon [11] discovered a new aldehyde reductase gene, *ari1*/YGL157W, from *S. cerevisiae,* NRRL Y-12632, which is a NADPH-dependent aldehyde reduction enzyme (Saccharomyces Genome Database http://www.yeastgenome.org/; accessed on 10 October 2021) with reduction activity for 14 aldehydes. The optimum temperature for this enzyme’s activity is 25 °C, the optimum pH is 7.0 and the protein molecular weight is 38 kDa. This enzyme is an intermediate member of the short-chain dehydrogenase/reductase superfamily and catalyzes Tyr169-XXX-Lys173, whose catalytic position requires four amino acids: Asn106, Ser131, Tyr169 and Lys173. The cofactor binding site for this enzyme is Gly11-XX-Gly14-XX-Ala17 near the N-terminal position [10,11].

In the present study, *ari1* was overexpressed in *S. cerevisiae* BCRC 21685. The engineered strain exhibited higher tolerance to aldehyde inhibitors, thereby enhancing the growth rate of *S. cerevisiae* and its ethanol production capacity. We investigated the growth patterns and degradation capacity of wild and engineered strains in YPD broth supplemented with furfural (20 mM) and HMF (60 mM). The engineered strains constructed in this study exhibited better responses at higher concentrations of HMF (60 mM).

## 2. Materials and Methods

### 2.1. Strains, Vectors and Media

*S. cerevisiae* (BCRC 21685) was purchased from the Bioresource Collection and Research Center, Food Industry Research and Development Institution, Shinchu, Taiwan. *Escherichia coli* TOP10F’ and the expression vector pGAPZαC were purchased from Novagen Inc. (Madison, WI, USA) and Invitrogen (Carlsbad, CA, USA) and served as the cloning host cell and expression vector, respectively.

Parent *E. coli* TOP10F’ and the Zeocin-resistant transformant were cultured, respectively, in a Luria–Bertani (LB) medium (10 g/L peptone, 10 g/L NaCl and 5 g/L yeast extract) and low salt Zeocin-LB plates (10 g/L peptone, 5 g/L NaCl, 5 g/L yeast extract and 25 mg/L Zeocin) at 37 °C. *S. cerevisiae* was maintained in a YPD medium (10 g/L yeast extract, 20 g/L peptone, 20 g/L dextrose) at 28 °C and 100 mg/L of Zeocin (Invitrogen Corp., Carlsbad, CA, USA) was used to select the engineered yeast. Vectors in the *E. coli* TOP10F’ and yeast genomic DNA were extracted using a Gene-spin miniprep plasmid purification kit (Protech Technology, Taipei, Taiwan) and Genomic DNA purification kit (BioKit, Miaoli, Taiwan).

### 2.2. Primers

Table 1 lists the primers used for *ari1* cloning, verification of the *ari1* gene insertion and quantitative real-time PCR in this study. The nucleotide sequences of the *ari1* gene (GenBank ID: NM_001181022.3) and *taf10* (GenBank ID: NM_001180474.3) (housekeeping genes used as the internal control) from *S. cerevisiae* were acquired from the NCBI website (National Center for Biotechnology Information, Bethesda, MD, USA).

### 2.3. Genetic Manipulation

The expression vector pGAPZαC carrying the *ari1* gene (pGAPZC-*ari1*) was constructed according to our previous study [12]. After being linearized with the *AvrII* enzyme, pGAPZC-*ari1* was transformed via electroporation according to the manufacturer’s instructions (MicroPulser electroporation apparatus, Bio-Rad Laboratories, Hercules, CA, USA). Zeocin-resistant colonies were selected and confirmed with PCR. The yeast carrying pGAPZC-*ari1* was named SCA, while the parent *S. cerevisiae* was named SC.

### 2.4. Expression of Recombinant Protein

Yeast was incubated in a YPD broth with shaking (150 rpm) at 30 °C for 9 h and pellets were collected for protein extraction [13]. Proteins were separated using SDS-PAGE according to Laemmli [14] and the target protein (aldehyde reductase) probed with the Anti-His tag was visualized with Western blotting [12].

### 2.5. Quantification of Gene Expression via Real-Time Reverse Transcription PCR (RT-qPCR)

To evaluate the *ari1* expression level under the pressure of furfural or HMF, yeast was cultured in fresh YPD broth (100 mL) containing 20 mM furfural or 60 mM HMF at 30 °C with shaking (150 rpm).

At the indicated time intervals, cells were collected and centrifuged at 10,000× *g* for 5 min. TRIzol reagent was applied to extract the total RNA (Life Technologies, Inc., Grand Island, NY, USA). An iScriptTM cDNA synthesis kit (BioRad, Hercules, CA, USA) was used to reverse transcribe RNA to DNA. Real-time PCR was performed with iQ SYBR Green Supermix (BioRad, Hercules, CA, USA) and quantified using a MiniOpticonTM system (BioRad, Hercules, CA, USA). Amplifications were performed under the following thermo-cycle conditions: pre-denaturation at 95 °C/3 min, 40 cycles at 95 °C/10 s and 57.8 °C/30 s and final extension at 95 °C/10 s. The gene expression level was analyzed using the BioRad CFX manager 2.1 software (BioRad, Hercules, CA, USA) and presented as the ratio of *ari1* to *taf10* [15].

### 2.6. Microorganism Growth

The growth of yeast was determined by measuring the OD_600_ or Colony Forming Units with YPD broth or YPD agar at the relevant time intervals.

### 2.7. Furfural and HMF Reduction

In a 500 mL baffled Erlenmeyer flask, one mL of log-growth culture was adjusted to an OD_600_ value of 0.3 and added to 100 mL of YPD broth containing 20 mM furfural or 60 mM HMF, and incubated at 30 °C with shaking at 150 rpm. After centrifugation at 10,000× *g* for 10 min, the supernatant was collected and filtered through a 0.45 mm membrane. To determine furfural and HMF concentrations, the HPLC system was equipped with a refractive index detector and ICSep ICE-COREGEL-87H3 column (Transgenomic, Omaha, NB, USA). The mobile phase was 5 mM H_2_SO_4_ with a flow rate of 0.8 mL/min [16]. The reduction of furfural and HMF was expressed as the decrease in furfural and HMF concentrations in the growth broth over time.

### 2.8. Ethanol Productivity

The log-growth yeast (1 mL, 3 × 10^7^ cells/mL) was inoculated into 100 mL YP broth (10 g/L yeast extract, 20 g/L peptone) containing 10% glucose. The cells were incubated statically at 30 °C and 1 mL of liquid was withdrawn at each indicated time interval. Gas chromatography was performed according to the procedure in [17].

## 3. Results

### 3.1. Molecular Characteristics

**Figure 1:**
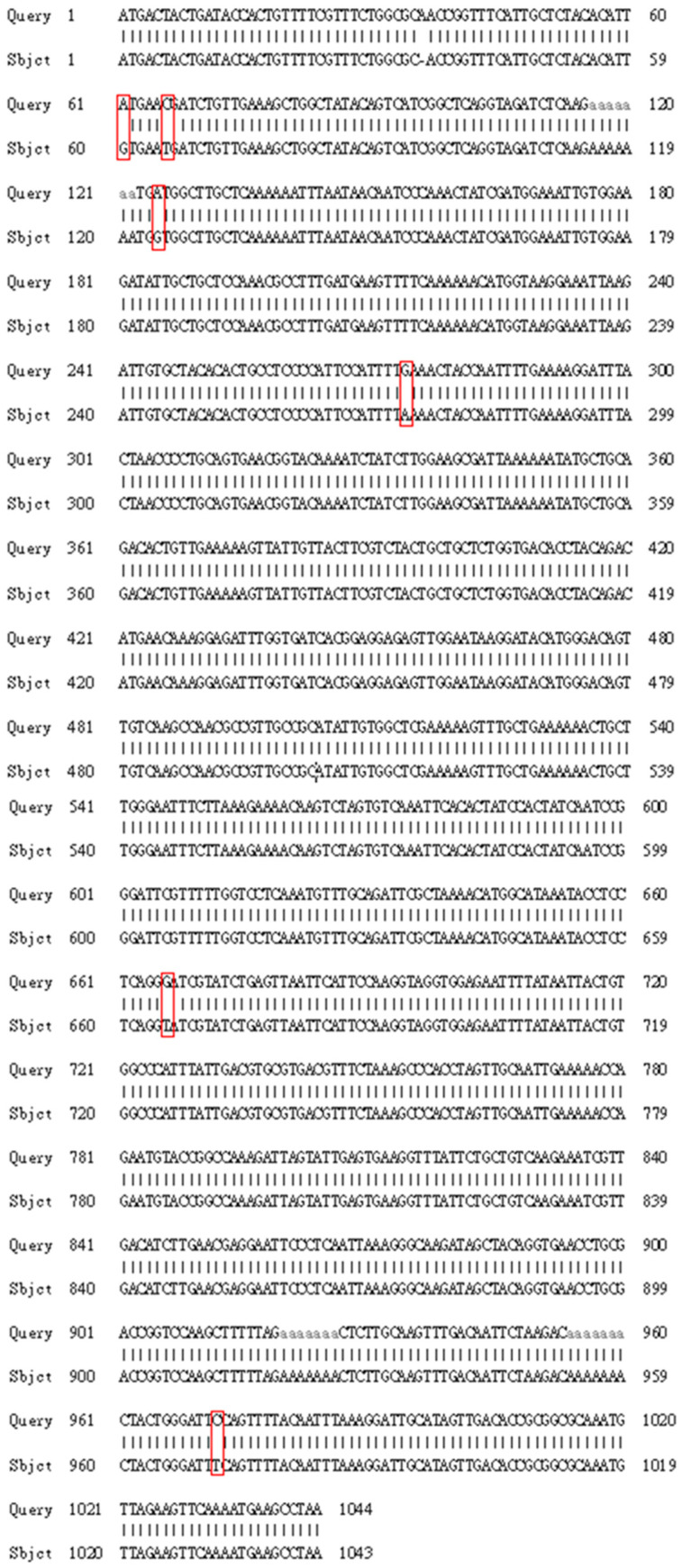
Comparison of the *ari1* DNA sequence for *ari1* obtained in this study with that published by Liu et al., 2009. Query: The *ari1* DNA sequence published by Liu et al., 2009. Subject: The ari1 DNA sequence from this study. It is shown that the DNA sequence of this study is different from that of Liu et al.

**Figure 2:**
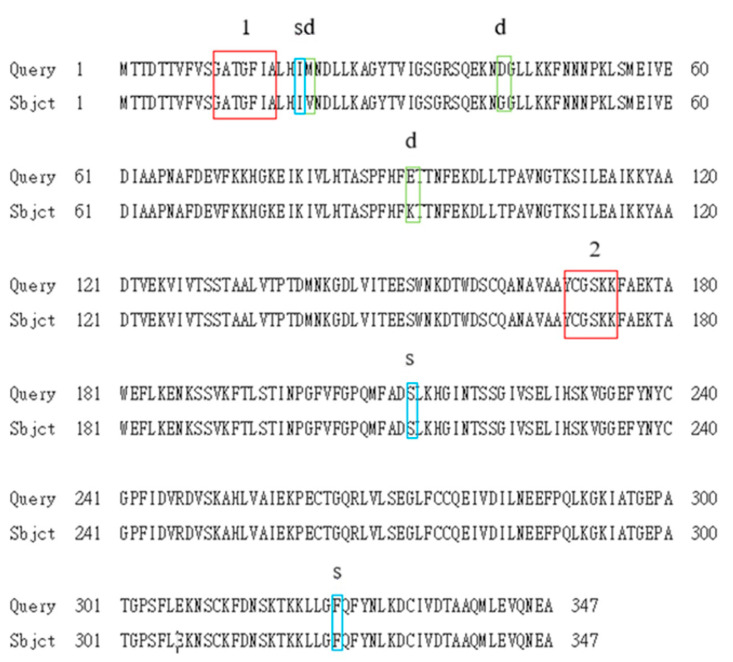
Comparison of the amino acid sequence for the ARI protein obtained in this study with that published by Liu et al., 2009. Query: The ARI amino acid sequence published by Liu et al., 2009. Subject: The ARI amino acid sequence in this study. ^1^ Cofactor binding site, ^2^ Active site, ^s^ DNA sequences were different from those of Liu et al. but their amino acids were the same. ^d^ Both DNA sequences and amino acids were different from those of Liu et al., 2009.

An *ari1* gene with 1044 bp was cloned in this study. This gene showed 99% similarity to the *ari1* open reading frame of *S. cerevisiae* S288c (NC-001139.8) listed in the NCBI gene bank (Figure 1). The aldehyde reductase expressed by the genetically recombined *S. cerevisiae* was found to have a total of six amino acids which were different from the protein sequence of the aldehyde reductase expressed by *S. cerevisiae* S288c (Figure 2). Fortunately, none of these amino acids were located in the enzyme active region (Tyr169-Lys173) or cofactor binding site (Gly11-Ala17).

### 3.2. Expression of Aldehyde Reductase

**Figure 3:**
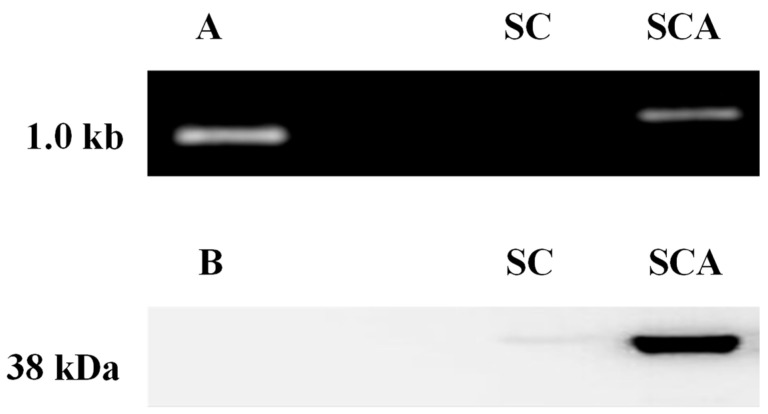
(**A**) PCR confirmation of the recombinant expression vector pGAPZC-ari1 in SC and SCA. (**B**) Overexpression of ARI in SC and SCA. SC (*S. cerevisiae*) and SCA (*S. cerevisiae* with *ari1* gene overexpression).

A DNA fragment appeared in the position at about 1.0 kb, which corresponds to the *ari1* gene (1044 bp was expected) in SCA (Figure 3A), whereas no PCR product was generated in the parental strain (SC). The result of Western blotting probed with an anti-His label (Figure 3B) confirmed that the SCA-expressed protein was in the range of 35~48 kDa, which matches the molecular weight of aldehyde reductase reported by Moon and Liu [18]. Again, SC did not produce the target protein band. The results of PCR and Western blot confirmed that *ari1* was successfully recombined and expressed in SCA.

### 3.3. Furfural Tolerance

**Figure 4:**
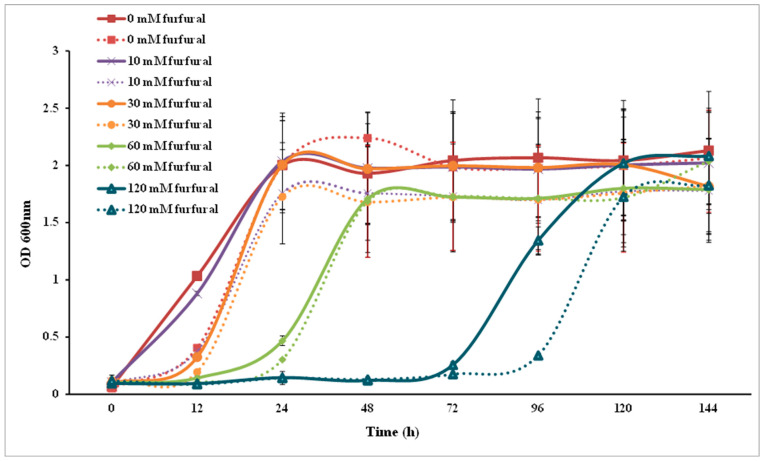
Furfural tolerance analysis of *Saccharomyces cerevisiae.* Solid lines represent engineered strains; dashed lines represent wild strains.

**Figure 5:**
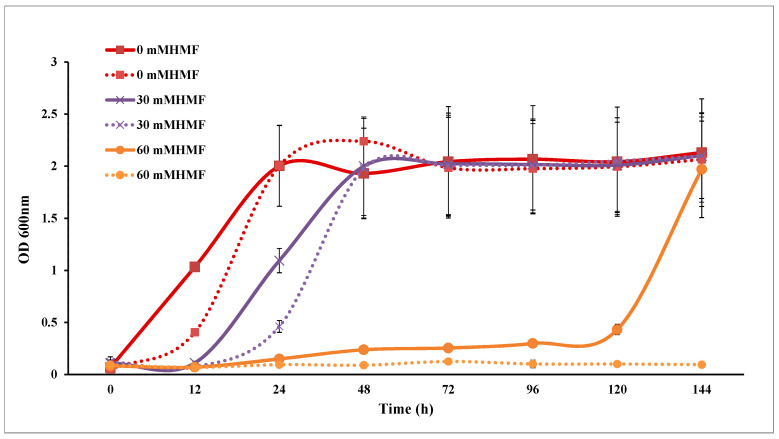
HMF tolerance analysis of *Saccharomyces cerevisiae*. Solid lines represent engineered strains; dashed lines represent wild strains.

Both SC and SCA were inhibited by furfural and HMF. The maximum OD_600_ values of SC and SCA were not affected, while their lag phases were extended in a dose-dependent manner (Figure 4 and Figure 5). The level of growth inhibition was greater in SC than in SCA.

### 3.4. ari1 Gene Expression

**Figure 6:**
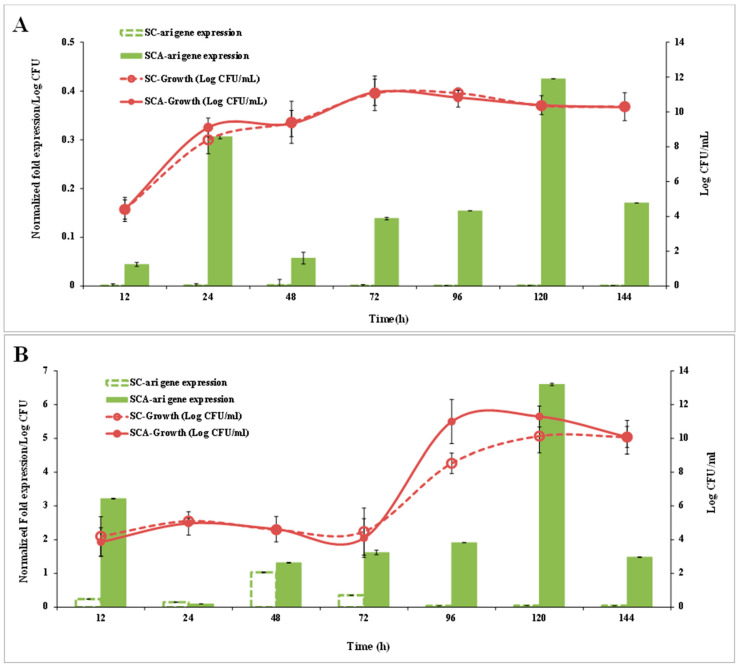

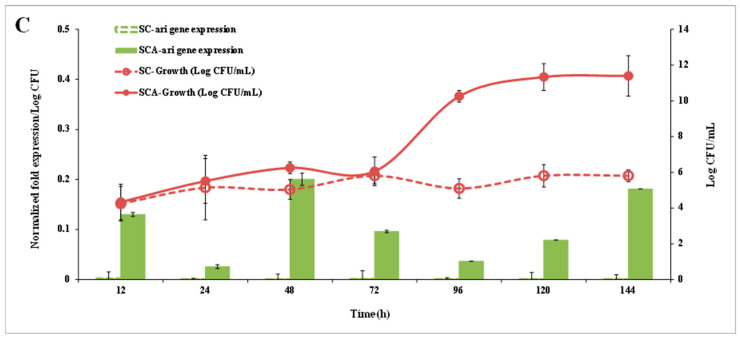
Ari gene expression (bar chart) and cell growth (graph) of SCA (filled symbol) and SC (open symbol) on the YPD medium (**A**), YPD + 20 mM furfural (**B**) and YPD + 60 mM HMF (**C**). SC (*S. cerevisiae*) and SCA (*S. cerevisiae* with *ari1* gene overexpression).

SC did not show *ari1* gene expression within 144 h of culturing in the YPD broth, while SCA showed *ari1* gene expression after 24 h of culturing. The expression of *ari1* in SCA was highest at 120 h-4.4-fold higher than that in SC. Although the growth patterns of SC and SCA were not significantly different, the expression of the *ari1* gene was higher in SCA than that in SC (Figure 6A).

Figure 6B,C show the *ari1* gene expression and growth performance of SC and SCA in the YPD broth supplemented with 20 mM furfural or 60 mM HMF, respectively. When YPD contained 20 mM furfural, the expression of *ari1* in SCA was higher than that in SC after 12 h of culturing and the expression of *ari1* in SCA was the highest after 120 h of culturing (74 times higher than that of SC). Both SC and SCA entered the logarithmic growth phase after 72 h of incubation; however, SCA showed the highest growth at 96 h of incubation (Figure 6B). In the presence of 60 mM HMF, the expression of *ari1* in SCA increased from 1.2-fold to 2-fold over an incubation period of 48~144 h. However, expression of the *ari1* gene in the parental strain remained below 0.008-fold. On the other hand, SCA entered the logarithmic growth phase after 72 h of culturing, but when there was a large amount of HMF (60 mM) in the medium, the SC did not show significant growth (Figure 6C).

### 3.5. Furfural and HMF Reduction Capacities

**Figure 7:**
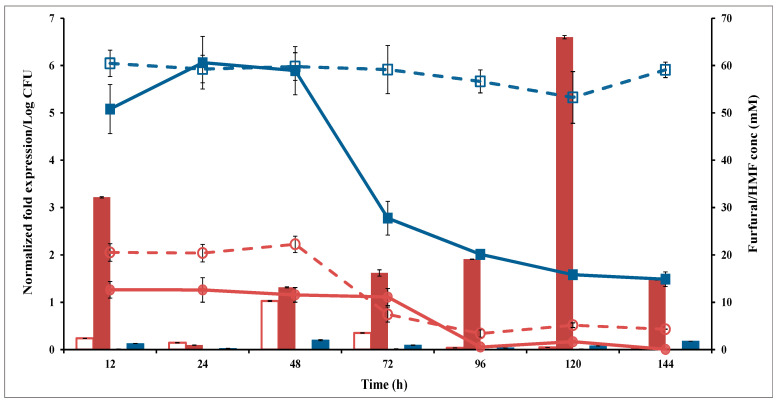
Ari gene expression (bar chart) and aldehyde degradation (line chart) of SCA (filled symbol) and SC (open symbol) in the presence of 60 mM HMF (Blue color) or 20 mM Furfural (Red color) on the YPD medium. SC (*S. cerevisiae*) and SCA (*S. cerevisiae* with *ari1* gene overexpression).

SC and SCA were independently incubated in the YPD broth and challenged with furfural (20 mM) or HMF (60 mM) to compare their abilities to reduce these compounds (Figure 7). SCA completely reduced furfural (20 mM) within 96 h; however, 4.3 mM furfural remained after SC was incubated for 144 h. SCA reduced HMF (60 mM) to 27 mM and 15 mM after 72 h and 144 h of incubation, respectively, whereas SC was unable to degrade HMF.

### 3.6. Ethanol Production Capacities

**Figure 8:**
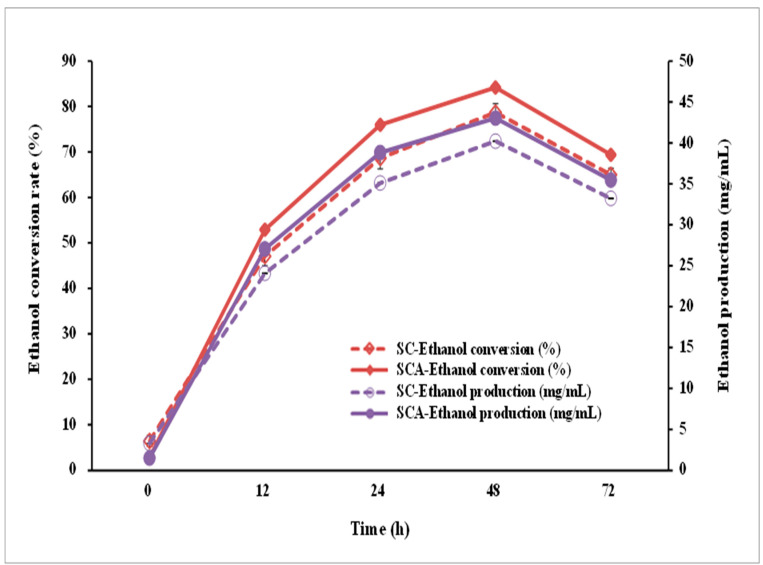
Ethanol conversion rate and production using SCA (filled symbol) and SC (open symbol) in the presence of 10% glucose on the YPD medium. SC (*S. cerevisiae*) and SCA (*S. cerevisiae* with *ari1* gene overexpression).

To evaluate the ethanol-producing capacity of the engineered strain, SCA was incubated in a high-glucose YPD broth (containing 10% glucose). After 48 h of incubation, both SC and SCA produced the largest amount of ethanol, with ethanol concentrations of 40.21 and 43.03 mg/mL, respectively (Figure 8); the conversion rates were 78.69 ± 1.88% and 84.21 ± 2.13%, respectively. SCA can more efficiently produce ethanol than its parent strain (SC).

## 4. Discussion

The bioconversion of lignocellulosic material to ethanol is a feasible technology based on inexpensive raw materials [1]; the main operations involve pretreatment, hydrolysis, fermentation and distillation [3]. The major components of lignocellulosic biomass are cellulose, hemicellulose and lignin. The relative amounts of these components vary among the different sources of lignocellulosic biomass. For example, plant cell walls contain 30–45% cellulose, 20–30% hemicellulose and 20–35% lignin [19]. Pretreatments, such as acid and alkaline hydrolysis, are commonly used to release hemicellulose (major) and lignin (minor) to facilitate enzyme access and ultimately increase ethanol production. Acid-pretreated hemicellulose is easily degraded into its constituent sugar units (mainly xylose, mannose, arabinose and glucose [20]), while fructose, arabinose, rhamnose, galactose, glucose and xylose were detected in the lignin hydrolysate [21]. However, monosaccharides can be converted to furan derivatives upon heating in acidic solutions, in which pentoses are dehydrated into furfural and hexoses are dehydrated into HMF [7].

Furfural and HMF are the most representative inhibitors of yeast growth [5,7]. Researchers have reported that these aldehydes can damage DNA, hinder RNA and protein synthesis and reduce enzymatic activity, thereby inhibiting cell growth [6,19,20,21,22,23].

Likewise, the multiple dehydrogenases/reductases in *S. cerevisiae* are capable of reducing furfural and HMF to their corresponding, less toxic alcohols [11,24]. Studies have shown that the overexpression of dehydrogenase/reductase genes (*ari1*, *adh7*, *ald6* and *adh6*) increases enzyme activities for furfural and/or HMF reduction while increasing the tolerance of yeast to inhibitors [14,15,16,17]. Liu and Moon [11] showed that *S. cerevisiae* NRRL Y-12632, which overexpressed the aldehyde reductase gene, was not only more tolerant to furfural (20 mM) and HMF (40 mM) than the wild type but also more easily recovered and subject to better growth. Strain improvement played important roles in cell viability and ethanol production under several different stress conditions.

Consistent with the above-mentioned factors, the engineered strain SCA (overexpression of *ari1* gene) constructed in this study showed greater tolerance to HMF and furfural and consequently increased ethanol production. SCA was more tolerant to furfural and HM than SC and increased resistance to HMF to a greater extent than furfural. Even though the *air1* expression in SCA incubated with 60 mM HMF was not as good as that in 20 mM furfural, the level of expression was sufficient to remove almost 100% of HMF.

Without the presence of HMF or furfural, the growth of SC and SCA was almost the same. Studies have shown that HMF is less toxic to yeast than furfural [25], so we compared the effects of higher HMF concentrations on yeast to those of lower furfural concentrations. According to Fenske et al. [23], the concentrations of furfural and HMF are approximately 0.01 g/L (0.1 mM) in corn stover, switchgrass and poplar pre-hydrolysates. Almeida et al. [22] reported that the HMF concentrations in spruce hydrolysate can vary from 2.0 to 5.9 g/L (15.8 and 46.6 mM), depending on whether one-step- or two-step-dilution acid hydrolysis is performed. The HMF concentrations are around 1 g/L (10 mM). A recent study reported by Erkan et al. [24] showed that 1 mM furfural decreased the ethanol yield by 10% when using *Saccharomyces cerevisiae*. Furfural is more toxic to *S. cerevisiae*. Based on the above literature, in this study 20 mM furfural and 60 mM HMF were used to analyze the *air1* gene expression levels when simulating the conversion process of lignocellulosic biomass. 

SC was found to overcome the toxicity of 20 mM furfural after 72 h incubation and subsequently showed growth. However, the growth rate was slower compared to that of SCA. On the other hand, 60 mM HMF was toxic to SC and caused SC to cease growth for 144 h. Inhibition with HMF and furfural exhibited distinct patterns on SC: 20 mM furfural increased the doubling time during logarithmic growth and decreased the amount of total biomass, while 60 mM HMF increased the lag phase to 144 h, the end of the experiment time. Both decreased the biomass or prolonged the lag phase under the stress of furfural and HMF, respectively, suggesting that the cells had difficulty adapting to the stress conditions. In terms of furfural and HMF’s reduction capacities, SCA significantly expressed *ari1* and reduced HMF from 60 to 29 mM under 72 h incubation, which allowed SCA to overcome the inhibitory effect of HMF. SC exhibited the ability to reduce furfural when cultured for 72 h, which was why SC did not show a delay in the lag phase but did present a decrease in biomass. 

At the same concentration levels, yeast strains are more sensitive to inhibition with furfural than that with HMF [25]. The effects of furfural and 5-hydroxymethylfurfural (5-HMF) on oxidative metabolism and fermentation were also investigated using *Candida guilliermondii* and *S. cerevisiae*, respectively. When added to the medium at a concentration of 0.2%, furfural was found to be a strong inhibitor of both functions, whereas a smaller dose of furoic acid was detected in the supernatant of the *C. guillermondii* medium. The inhibitory effect of 5-HMF on fermentation and growth was weak and no metabolite was detected in the supernatant. The results showed that the metabolic pathways of furfural and 5-HMF are different depending on whether they enter the fermentation pathway or the oxidative pathway in the yeast strains studied [6].

## 5. Conclusions

In this study, an inhibitor-tolerant yeast strain was constructed. This strain showed enhanced abilities to reduce furfural and/or HMF through overexpression of the *ari1* gene, thus indicating higher cell viability in the environment containing aldehydes.

## Figures and Tables

**Table 1:** Oligonucleotides used in this study.

Primer	Sequence 5′-3′	Purpose
Forward	5′TCGTTCGAAAAAATGGCGACTACTGATACCACTGTTTTCGTTTCTG-3′	*ari1* cloning
Reverse	5′TCACTCGAGTTAGGCTTCATTTTGAACTTCTAACATTTGCGCCGC-3′	
VF	TTCGAAAAAATGGGTACTAC	Verification of *ari1* gene insertion
VR	AGTGATGGTGATGGTGATGG	
qAri1-F	TTGTGCTACACACTGCCTCC	Quantitative real-time PCR
qAri1-R	CGTTCACTGCAGGGGTTAGT	
qTaf10-F	TCCAGGATCAGGTCTTCCGT	
qTaf10-R	TGTCCTTGCAATAGCTGCCT	

Underlined text indicates *BstB1* and *Xho1* recognition sequences in the forward and reverse primers, respectively; highlighted text indicates the Kozak consensus sequence; ATG and TTA represent the start and stop codons.

## Data Availability

The data presented in this study are available on request from the corresponding author.
